# A Study of Cecal Ligation and Puncture-Induced Sepsis in Tissue-Specific Tumor Necrosis Factor Receptor 1-Deficient Mice

**DOI:** 10.3389/fimmu.2019.02574

**Published:** 2019-11-08

**Authors:** Jolien Vandewalle, Sophie Steeland, Sara Van Ryckeghem, Melanie Eggermont, Elien Van Wonterghem, Roosmarijn E. Vandenbroucke, Claude Libert

**Affiliations:** ^1^VIB Center for Inflammation Research, Ghent, Belgium; ^2^Department of Biomedical Molecular Biology, Ghent University, Ghent, Belgium

**Keywords:** sepsis, inflammation, TNF, TNFR1, cecal ligation and puncture, lipopolysaccharide

## Abstract

Sepsis is a complex syndrome resulting from a dysregulated immune response to an infection. Due to the high prevalence, morbidity, and mortality, there is a lot of interest in understanding pathways that play a role in sepsis, with a focus on the immune system. Tumor necrosis factor (TNF) is a pleiotropic pro-inflammatory cytokine and a master regulator of the immune system but clinical trials with TNF blockers in sepsis have failed to demonstrate significant protection. Since TNF stimulates two different receptors, TNF receptor 1 (TNFR1) and TNFR2, pan-TNF inhibition might be suboptimal since both receptors have opposite functions in polymicrobial sepsis. Therefore, we hypothesized that TNF has a dual role in sepsis, namely a mediating and a protective role, and that protection might be obtained by TNFR1-specific inhibition. We here confirmed that TNFR1^−/−^ mice are protected in the sterile endotoxemia model, whereas TNFR1 deficiency did not protect in the cecal ligation and puncture (CLP)-induced polymicrobial sepsis model. Since whole body TNFR1 blockage might be deleterious because of the antibacterial function of TNF/TNFR1 signaling, we focused on the potential devastating role of TNF/TNFR1 signaling in specific cell types. We were interested in the gut epithelium, the endothelium, and hepatocytes using conditional TNFR1^−/−^ mice, as these cell types have been shown to play a role in sepsis. However, none of these conditional knockout mice showed improved survival in the CLP model. We conclude that cell-specific targeting of TNFR1 to these cell types has no therapeutic future in septic peritonitis.

## Introduction

Sepsis is an acute condition resulting from a dysregulated host response to an infection. It is a major cause of morbidity and mortality among hospitalized patients and the leading cause of death among patients in intensive care units (ICUs). It is thought that up to 19 million cases arise yearly, and that the overall mortality is about 20–25%, reaching up to 40% in case of septic shock. Sepsis patients are usually treated with antibiotics, resuscitation, and organ function support. But despite huge investments over the last 30–40 years into sepsis, no therapeutics have reached the bedside (1).

Although sepsis is considered as an inflammatory condition, numerous clinical trials targeting inflammatory mediators have consistently failed in human sepsis patients. The reasons for these failures are believed to reside in the complexity of sepsis at the level of the patient, the infectious agent and the immune response (2). In the past, the host's immune response to an external pathogen has been divided in two phases. An early phase typically characterized by an overwhelming immune response leading to an excessive production of cytokines, followed by a compensation mechanism through which the patient enters an immunosuppressive state (3). This is however not a systemic phenomenon as the location of the immune cells greatly influence their functional status. The concepts of “leukocyte reprogramming” or “trained immunity” are a more appropriate way of qualifying events associated with the anti-inflammatory response. The ultimate goal of these mechanisms is probably aimed at preventing an excessive pro-inflammatory response, while maintaining the defense mechanisms (4).

The pro-inflammatory response is driven primarily by cytokines and inflammatory mediators released by the innate immune system. The pro-inflammatory cytokine tumor necrosis factor (TNF), has long been considered as a top-candidate mediator in sepsis patients and animals (5). Indeed, TNF inhibition using antibodies protected in a model of Gram-negative infection with live *Escherichia coli* (6). Conversely, injection of recombinant TNF causes systemic inflammation in humans and animals (7). However, up until now, 18 different clinical trials using TNF inhibitors have been performed in sepsis patients with very minimal impact on the survival rates (8).

TNF-induced lethal systemic inflammation was demonstrated to depend entirely and exclusively on TNFR1 (9, 10). Since TNF is able to bind and activate two different receptors, namely TNF receptor 1 (TNFR1), generally considered as the inflammation-mediating receptor, and TNFR2, considered as the immune modulating receptor (11), we and others have argued that TNF/TNFR1 inhibition should be considered in sepsis rather than full TNF antagonism (12, 13).

To investigate this hypothesis, we applied a highly validated mouse model of septic polymicrobial peritonits, namely cecal ligation and puncture (CLP) (14), using conventional as well as cell-specific TNFR1-deficient animals. We focused on cell-specific deletion of TNFR1 in intestinal epithelial cells (IECs), endothelial cells and hepatocytes. Our results reveal that neither whole body nor cell-specific TNFR1 deficiency lead to significant improvement of the survival rates upon CLP-induced sepsis implicating that TNFR1 targeting is not a suitable treatment strategy in sepsis.

## Results

### TNFR1 Plays a Mediating Role in Lethal Endotoxemia

**Figure 1:**
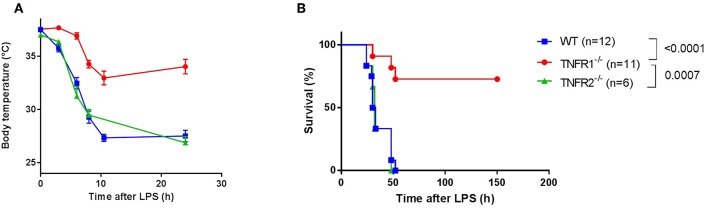
Study of the effect of general TNFR1^−/−^ in LPS-induced endotoxemia. **(A,B)** C57BL/6J wild type (WT) (*n* = 12), TNFR1^−/−^ (*n* = 11), and TNFR2^−/−^ mice (*n* = 6) were intraperitoneally (i.p.) injected with a lethal dose of LPS (6.25 mg/kg). Body temperature **(A)** and lethality **(B)** were recorded. The last animals succumbed 52 h after challenge.

In order to confirm the specific role of TNFR1 in acute lethal endotoxemia, we investigated the response of whole body TNFR1^−/−^ mice and TNFR2^−/−^ mice to a single intraperitoneal injection of lipopolysaccharide (LPS), and compared it with the response of Wild Type (WT) mice. This injection leads to a lethal response, and we studied hypothermia and lethality over a period of 150 h (no later deaths occurred). In agreement with previous studies (15–17), we found that TNFR1^−/−^ mice were significantly more protected to both hypothermia and lethality compared to WT (*p* < 0.0001) and TNFR2^−/−^ animals (*p* = 0.0007). In contrast, TNFR2^−/−^ animals displayed no protection (Figures 1A,B).

### Full TNFR1^−/−^ Mice Are Not Protected in the CLP Model

**Figure 2:**
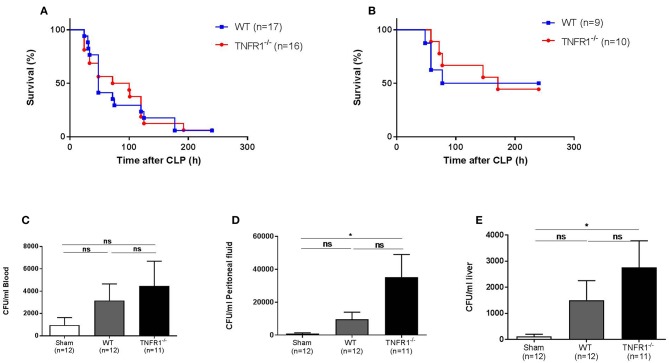
Study of the effect of general TNFR1^−/−^ in the cecal ligation and puncture model. **(A,B)** Male WT (*n* = 17) and TNFR1^−/−^(*n* = 16) were subjected to lethal and sublethal CLP. All mice were treated with antibiotics 10 and 24 h after the CLP procedure. Survival was monitored for 10 days. **(C–E)** Six hours after CLP, blood **(C)**, peritoneal lavage fluid **(D)**, and liver homogenate **(E)** were collected from sham-operated (*n* = 12) and lethal CLP WT (*n* = 12) and TNFR1^−/−^ (*n* = 11) mice. Total bacterial counts were determined and expressed as colony-forming units (CFU) per ml. *p ≤ 0.05 and ns, not significant.

As the LPS-induced model is a sterile model, this model does not represent real polymicrobial sepsis. Therefore, we studied the role of TNFR1 in the cecal ligation and puncture (CLP) model. This is a more reliable and well-validated model for sepsis, and the model is considered being the golden standard of human peritonitis (14). First, we investigated the survival rate of whole body TNFR1^−/−^ mice subjected to CLP, and compared it with WT mice. Figure 2A displays the general outcome of two independent experiments, where we did not find any resistance in TNFR1^−/−^ mice compared to WT controls in this model. The experiment was repeated using a sublethal CLP, but again no difference in mortality between WT and TNFR1^−/−^ mice was observed (Figure 2B). Since TNF and TNFR1 have been found to be important in antimicrobial defenses, a general absence of TNFR1 might be a sensitizing factor in sepsis. To investigate this role in polymicrobial sepsis, we determined the bacterial counts in blood, peritoneal exudate and liver homogenate, 6 h after CLP in TNFR1^−/−^ and WT mice, and compared it with the counts in sham-operated mice. In blood, we could not detect any significant increase in colony forming units (CFU) when sham-treated mice were compared with WT or TNFR1^−/−^ mice that were subjected to CLP (Figure 2C), although we detected a slight trend toward higher bacterial counts in the TNFR1^−/−^ mice. In the peritoneal exudate and liver homogenate, the bacterial counts in CLP-subjected TNFR1^−/−^ mice were significantly higher than in sham-operated mice, whereas we could not detect a significant increase in CLP-subjected WT mice compared to sham mice (Figures 2D,E). Based on the protection of TNFR1^−/−^ mice in sterile endotoxemia, but clear lack of protection in an infectious sepsis model, it could be suggested that TNF/TNFR1 signaling pathway is crucial in the clearance of bacteria upon sepsis and thus has an indispensable function to cope with these bacteria.

### Full TNFR1^−/−^ Mice Exhibit Reduced LDH and Vascular Permeability in the CLP Model but Are Not Protected Against Intestinal Permeability or Liver Damage

**Figure 3:**
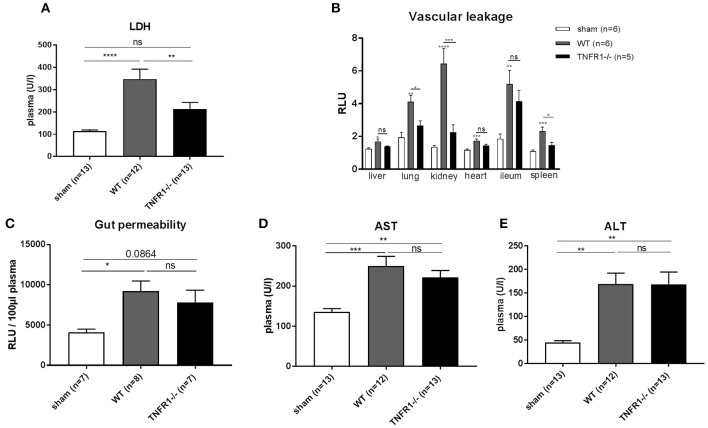
Study the role of TNFR1 on vascular, intestinal and liver damage in the cecal ligation and puncture model. Male WT and TNFR1^−/−^ were subjected to lethal CLP and following parameters were measured 8 h after CLP. **(A)** LDH was measured in plasma (*n* = 12/13). **(B)** Vascular permeability shown as relative light units (RLU) of FITC-dextran in liver, lung, kidney, heart, ileum, and spleen after iv injection (*n* = 5/6). **(C)** Gut permeability shown as RLU of FITC-dextran in plasma after gavage (*n* = 7–8). Aminotransferase levels **(D)** AST and **(E)** ALT levels were determined in plasma (*n* = 12–13). ****p < 0.0001, ***p < 0.001, **p < 0.01, *p ≤ 0.05 and ns, not significant.

TNFR1 plays a mediating role in the sterile LPS model by inducing injury in the liver (18), the gut barrier (19), and the endothelium (20). Therefore, we studied the role of TNFR1 in these organs in the CLP model. We detected decreased release of lactate dehydrogenase (LDH) in plasma of the full TNFR1^−/−^ mice 8 h after CLP (Figure 3A), indicating reduced cell damage or cell death. Since vascular hyperpermeability is a major feature in SIRS and sepsis, we assessed the vascular permeability in full TNFR1^−/−^ and WT mice in the CLP model. FITC-dextran was injected i.v. 5 h after CLP and vascular leakage was measured in different organs (Figure 3B). CLP leads to a significant increase in vascular permeability in all the organs tested (liver, lung, kidney, heart, ileum, and spleen). Interestingly, there was a constant tendency of reduced vascular leakage in TNFR1^−/−^ mice and this was significant in the lungs, kidney and spleen. Next, increased intestinal permeability is also believed to play a role in SIRS and sepsis. To investigate the role of TNFR1 in CLP induced intestinal permeability, FITC-dextran was administered by oral gavage 3 h after CLP and leakage of FITC-dextran from the gut to the blood was measured. Intestinal permeability was elevated after CLP, but no difference could be detected between the two genotypes (Figure 3C). Lastly, liver damage has been shown to occur in both SIRS and sepsis. Excessive TNF leads to liver damage and elevated transaminase levels in circulation. Alanine aminotransferase (ALT) and aspartate aminotransferase (AST) were significantly increased 8 h after CLP in both genotypes, but were not significantly different between CLP-subjected TNFR1^−/−^ and WT mice (Figures 3D,E).

### TNFR1 Conditional Intestinal Epithelium, Endothelial, and Hepatocyte Knockout Mice Are Not Protected in CLP

**Figure 4:**
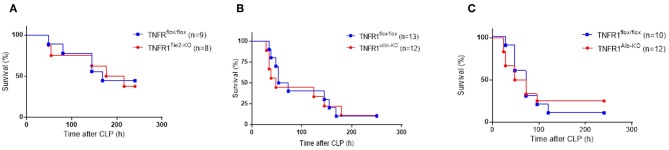
Study on the role of TNFR1 in endothelium, gut epithelium, and hepatocytes using conditional knock-out mice in the cecal ligation and puncture model. Male TNFR1^flox/flox^ and cell-specific conditional TNFR1^−/−^ mice were subjected to CLP. Survival was monitored for 10 days in **(A)** endothelium (TNFR1^Tie2−KO^) (*n* = 8), **(B)** gut epithelium (TNFR1^Villin−KO^) (*n* = 12) and **(C)** hepatocyte (TNFR1^Alb−KO^) specific (*n* = 12) knockout mice, and compared with TNFR1^flox/flox^ mice (*n* = 9, 13, and 10, respectively).

It is possible that different cellular sources of TNFR1 mediate different effects, and in order to distinguish the different functionalities of TNF/TNFR1 signaling in different cell types, we generated tissue-specific TNFR1-deficient mice by crossing TNFR1^flox/flox^ mice with three different cre lines. We reasoned based on literature that the most obvious candidate cells that would suffer the most from TNF in this model and sepsis in general would be the intestinal epithelial cells (IECs), endothelial cells and hepatocytes (21–23). After obtaining these cell-specific conditional TNFR1^−/−^ mice using Villin-, Tie2-, or albumin cre lines, respectively, we subjected them to the CLP-induced sepsis model and compared their response with that of the TNFR1^flox/flox^ control mice. As shown in Figures 4A–C, ablation of TNFR1 in none of these tissues has a beneficial effect on survival, and is therefore not of interest for therapeutic cell-specific antagonism.

## Discussion

In 2016, a refined definition of sepsis has been accepted by the community as the Third International Consensus Definition for Sepsis and Septic Shock (Sepsis-3) (1). Now, sepsis is defined as a life-threatening organ dysfunction caused by a dysregulated host response to infection. In contrast to the Sepsis-2 definition, this definition no longer refers to inflammation as an essential pathway in sepsis (1). One of the reasons of this adjustment is that the clinical trials testing inhibitors of inflammatory cytokines have failed to provide significant survival benefits, despite promising results in pre-clinical models (2). This recurrent problem makes scientists wonder what the community has missed or overlooked over the last decades. There has been tremendous discussion about the use of pre-clinical mouse models for studying acute inflammation in human diseases. In 2013, Seok et al. identified a poor correlation of the human and murine gene signatures in leukocytes after acute inflammatory stresses, such as burn, trauma and endotoxemia, thereby questioning the mouse as a research model (24). This conclusion was however challenged by a reanalysis of these same data but with restriction to the preselected genes that significantly changed in both human and mice after insult (25). The approach of the latter study is however controversial as pre-selection of genes introduces bias (26). Yet, there are substantial studies where animal models did predict the human response to sepsis. Anti-TNF studies are life-saving in LPS-induced SIRS models, but fail in septic patients, an argument used in the debate against murine models. However, anti-TNF antibodies used in a clinically more relevant model of sepsis, the CLP model, also failed to show any protection, resembling the human situation (27). This notion supports the need of using the most relevant animal models and preferably testing in multiple types of infectious disease models (CLP-induced peritonitis, pneumoniae).

Tumor necrosis factor (TNF) is a pleiotropic pro-inflammatory cytokine and plays an important role in numerous inflammatory diseases. TNF inhibiting drugs are powerful and popular, and they revolutionized the treatment of rheumatoid arthritis, Crohn disease, and psoriasis. However, TNF-inhibiting biologicals can lead to a number of side effects (28). TNF can bind and activate two different receptors, TNFR1 and TNFR2. TNFR2 has been shown to be an immune-regulatory receptor, which stimulates development of anti-inflammatory regulatory T cells. Consequently, some authors believe that the side-effects associated with the TNF inhibitors come from the abrogation of the TNF/TNFR2 signaling in addition to the TNF/TNFR1 signaling (13). Studies using TNFR1^−/−^ mice and TNFR2^−/−^ mice, as well as TNFR1-specific inhibitors have confirmed that the aforementioned hypothesis might be true in some cases (13). For example, in multiple sclerosis, TNF inhibition has no therapeutic effect and could even lead to disease exacerbations, whereas TNFR1 inhibition clearly has protective effects, as we recently discovered using the Nanobody-based TNFR1-specific inhibitor, called TNF Receptor One Silencer (TROS) (29). Furthermore, we also showed therapeutic effect of TNFR1 inhibition using TROS in two different mouse models of Alzheimer's disease (30).

The current study was designed using the same reasoning. TNF inhibition in sepsis patients has no obvious therapeutic effect and a meta-analysis of all trials seems to suggest only a minimal protective effect in the worst cases of septic shock (8). These trials were a huge disappointment, particularly since (i) early studies suggested promising effects of TNF-inhibiting antibodies in a baboon sepsis model with Gram-negative infection (6), (ii) TNF injections in animals and patients lead to very similar pathophysiological responses as those observed in sepsis (7), and (iii) several studies have found a link between polymorphisms in the TNF promoter and susceptibility for sepsis development (31).

A possible explanation for the failure of TNF inhibitors in sepsis might be linked to the TNFR2 inhibition (12). Ebach et al. showed that TNFR1^−/−^ mice show prolonged survival in the CLP model, whereas TNFR2^−/−^ mice had shortened survival. These data suggest that in sepsis, TNFR1 plays a mediating role and TNFR2 rather a protective role (12). Moreover, we recently reported that human sepsis patients display a significant increase in levels of soluble TNFR1 and *TNFR1* gene expression in blood and blood cells, respectively (17). Additionally, simultaneous inhibition of matrix metalloproteinase (MMP) 8 and TNFR1 protects against both endotoxemia and CLP-induced sepsis (17). In our study, we tested TNFR1^−/−^ mice in the CLP model, but no differential response of TNFR1^−/−^ mice in comparison to WT mice was seen, while TNFR1 deficiency did show protection in the endotoxemia model in agreement with other studies (9, 16). In contrast, one study showed even sensitization of TNFR1^−/−^ mice in the CLP model (32). Overall, the exact role of TNFR1 in sepsis remains unclear. The differences observed in these studies could result from different techniques used to induce sepsis. There is quite some variation in the CLP procedure resulting in different mortality and this could even contribute to different results (33). The percentage of cecum that is ligated and the number and size of punctures in the cecum are factors contributing to the severity of CLP. Additionally, antibiotics, the most important elements in the treatment of sepsis, are not always included in animal studies. For example, the study of Hildebrand et al. did not apply antibiotics, whereas in our study antibiotics were provided to correlate better with the human situation (32). Also the type of cecal bacteria involved in the pathogenesis could be responsible for different mortality and controversy in the CLP model. A recent paper showed that commensal bacteria in the phylum Proteobacteria can induce serum IgA, resulting in protection against CLP-induced sepsis (34).

A second explanation for the failures of anti-TNF drugs deals with the antimicrobial function of TNF. TNF knockout as well as TNFR1 knockout mice have proven to be very sensitive in infectious models, as the absence of TNF and TNFR1 leads to an inadequate innate immune response (13). When studying the number of colony forming units following CLP, we found a trend toward more contamination in blood of TNFR1^−/−^ mice compared to CLP-subjected WT mice. In the peritoneal cavity and liver, significantly higher levels of colony forming units were found in CLP-subjected TNFR1^−/−^ mice compared to sham-operated mice, and this was not the case for CLP-subjected WT mice. These data suggest that TNFR1 deficiency might undermine the antibacterial immunity, and that therefore protective effects are lost. It has been shown previously, that TNFR1^−/−^ mice, indeed, suffer from significantly less controlled bacterial infections, e.g., in models of Streptococcus or Citrobacter (35). This reflection formed the basis to investigate whether a depletion of TNFR1 on cells which are not involved in the antibacterial immunity in the CLP model might lead to improved survival rates. We chose to deplete TNFR1 in cells (i) which are known to respond strongly to TNF, for example after TNF injection (21) and (ii) which are believed to play a role in sepsis (21–23), namely IECs, endothelial cells and hepatocytes. These are cells that might be targeted by TNFR1-specific inhibitors, such as TROS, linked with for example Nanobodies that target specifically hepatocytes (asialoglycoprotein receptor) or endothelium (VCAM1).

Our results illustrate the susceptibility of three different organs toward organ damage in CLP-induced sepsis, namely liver, gut, and endothelium. During SIRS and sepsis, these organs are indeed known to be vulnerable for increased damage, eventually resulting in multiple organ failure. Intestinal damage accompanied by apoptosis and detachment of IECs are typical features of TNF-induced SIRS, leading to gut barrier leakiness. The gut has long been hypothesized to be “the motor” of critical illness. Former research in our lab has shown that intestinal TNFR1 plays a critical role in TNF-induced shock (21). Reduction of TNFR1 expression specifically in IECs mitigates TNF toxicity and this is linked with a reduction in TNF-induced intestinal permeability and systemic inflammation (21). However, Duprez et al. have shown that gut damage is not immediately linked to mortality in TNF-induced SIRS. They showed that Caspase 3^−/−^ mice are protected against gut damage, but these mice do not show improved survival (36). The other way around holds also true, since RIPK3^−/−^ mice and Necrostatin-1 (a necroptosis inhibitor) pretreatment of WT mice protect against TNF-induced lethality, but do not protect against gut damage. In contrast, liver damage was significantly reduced in these mice, indicating that liver damage might contribute to TNF mortality (36). We studied liver and gut damage in TNFR1^−/−^ and WT mice after CLP but could not find a difference in organ damage. This could explain the lack of protection in respectively TNFR1^AlbKO^ and TNFR1^villinKO^ mice. In contrast, vascular permeability was reduced in all organs tested in the full TNFR1^−/−^ mice. Specific deletion of TNFR1 in endothelial cells however did also not protect against CLP induced lethality. Our results could imply that cell-specific targeting of TNFR1 to one of these three cell types has no future in sepsis, however, a synergistic protection of TNFR1 in different organs together could possibly still result in reduced mortality. Furthermore, we studied the role of TNFR1 only in liver, gut and endothelium. However, LDH release was significantly lower in TNFR1^−/−^ mice after CLP. Since LDH is released into the bloodstream when tissues are damaged, it is plausible that TNFR1 plays a mediating role in other organs than the three we tested. The most significant difference in vascular permeability was seen in the kidneys. As acute kidney injury (AKI) occurs in 40–50% of the septic patients and increases the mortality risk 6- to 8-fold, it would be of interest to study the role of TNFR1 in the kidneys in the CLP model.

The data presented here suggest that TNFR1 is an essential mediator in endotoxemia, which is a mouse model of sterile SIRS, induced by lipopolysaccharides. In a mouse model of polymicrobial sepsis, induced by CLP, low grade inflammation is induced by slow release of cecal content (bacteria, yeast, molds, viruses etc.) into the peritoneum. This model is considered as a useful model of septic peritonitis, which accounts for ~30% of human sepsis cases (37). Based on the finding that organism-wide TNFR1-deficiency has no differential response in the CLP model, and based on the knowledge that TNFR1 is essential in antimicrobial resistance, we tested the hypothesis that retention of TNFR1 on peritoneal WBC, but depletion of TNFR1 on potential TNF-target cells, responding to the inflammatory properties of TNF, may uncouple the beneficial from the harmful effects of TNF in sepsis, as predicted in recent opinion pieces (38). Our data suggest that cell-specific inhibition of TNFR1 in hepatocytes, endothelium or intestinal epithelium provides no therapeutic benefit in septic peritonitis.

## Materials and Methods

### Mice

All mice were housed in specific-pathogen free conditions with 14–10 h of light and dark cycles and free access to food and water. We used 8–12 week old mice, females for endotoxemia experiments, and males for CLP experiments. Wild-type, TNFR1^−/−^ [generated by M Rothe (39)], and TNFR2^−/−^ animals (40), all on a pure C57BL/6J background were originally purchased at the Jackson Laboratories, and further bred in our animal house. TNFR1^flox/flox^ mice, which were a kind gift from Dr. G. Kollias (Alexander Fleming Biomedical Sciences Research Center, Vari, Greece), were crossed with Villin-, Tie2-, or albumin cre mice to generate, respectively, intestinal epithelial cell (IEC), endothelial cell or hepatocyte-specific TNFR1 conditional knockout mice (41).

### Endotoxemia Model

Female mice were injected intraperitoneally with a lethal dose of LPS [LD_100_ 6.25 mg/kg, *Salmonella enterica* (Sigma-Aldrich)] dissolved in sterile PBS to mimic endotoxin shock. Rectal body temperature (first 24 h are shown) and lethality was monitored and pooled from two independent experiments.

### Cecal Ligation and Puncture (CLP) Induced Polymicrobial Sepsis

The CLP procedure was performed according to the general guidelines (14). Briefly, male mice were anesthetized by isoflurane inhalation and a one-centimeter incision was made in the abdomen after which the cecum was exposed and ligated. This was followed by making two punctures in the cecum with a 21-Gauge needle for induction of a lethal CLP and one puncture with a 23-Gauge needle for induction of a sublethal CLP. The abdominal musculature and skin were closed with simple running sutures and metallic clips, respectively. Ten and 24 h after CLP, mice were injected intraperitoneally with an antibiotic cocktail containing ceftriaxone (25 mg/kg; Sigma-Aldrich) and metronidazole (12.5 mg/kg; Sigma-Aldrich) dissolved in 200 μl PBS. Rectal body temperature and lethality was monitored for 10 days and pooled.

### Determination of Bacterial Load

Blood was taken via cardiac puncture after anesthetization with a lethal mix of ketamine/xylazine and collected in EDTA-coated tubes. Peritoneal fluid was taken by peritoneal lavage with 4 mL of PBS containing 1 mM EDTA. 50 mg liver was isolated and homogenized in 600 μl PBS. Serial dilutions of blood, peritoneal fluid or liver homogenate were prepared in sterile PBS for plating on brain-heart-infusion agar plates. Plates were overnight incubated at 37°C. Viable counts of bacteria were expressed as colony-forming units per ml blood.

### LDH, ALT, AST in Blood

Eight hours after CLP or sham, blood was obtained via retro-orbital bleeding, sampled in EDTA-coated tubes and plasma was prepared (3,000 rpm, 20 min, 4°C). Plasma was diluted in 0.9% NaCl and biochemical analysis was performed by the University Hospital of Ghent.

### Gut Permeability Assay

Three hours after CLP or sham, 100 μl of 100 mg/ml FITC-dextran (4 kDa, Sigma-Aldrich) was gavaged. Five hours after gavage, blood was obtained via retro-orbital bleeding, sampled in EDTA-coated tubes and plasma was prepared (3,000 rpm, 20 min, 4°C). Plasma was diluted ½ in PBS and fluorescence was measured with a FLUOstar OMEGA plate reader (BMG Labtech, Germany).

### Vascular Permeability Assay

Six hours after CLP or sham, 25 mg/kg FITC-dextran (4 kDa, Sigma-Aldrich) was injected intravenous. One hour later, mice were anesthetized with a lethal mix of ketamine/xylazine and transcardially perfused with 0.2% heparin in PBS. A selected set of organs was isolated, cut in small pieces and shaken overnight in 100% formamide (Sigma-Aldrich) at 37°C to extract FITC-labeled dextran from the tissue. After 18 h incubation, the samples were centrifuged at 13,000 rpm for 15 min and supernatant was diluted 1/20 in PBS and fluorescence measured with a FLUOstar OMEGA plate reader (BMG Labtech, Germany). Fluorescence was corrected for weight of the samples and normalized to the lowest value.

### Statistics

Data are represented as mean ± SEM. *P*-values for survival curves were analyzed with a Log-rank test. *P*-values for all other experiments were analyzed with one-way ANOVA. Data of two independent experiments were pooled.

## Data Availability Statement

All datasets generated for this study are included in the article/supplementary material.

## Ethics Statement

All animal experiments were reviewed and approved by the ethical committee for animal welfare of the Faculty of Sciences Ghent University.

## Author Contributions

JV and SS contributed equally to the experimental work. JV wrote the manuscript. SV, ME, and EV assisted with some experiments. SS, RV, and CL supervised the experiments and the manuscript.

### Conflict of Interest

The authors declare that the research was conducted in the absence of any commercial or financial relationships that could be construed as a potential conflict of interest.
